# Identifying and Characterizing a Novel Peritrophic Matrix Protein (*Md*PM-17) Associated With Antibacterial Response From the Housefly, *Musca domestica* (Diptera: Muscidae)

**DOI:** 10.1093/jisesa/ieaa135

**Published:** 2020-12-21

**Authors:** Yu Wang, Jinzhi Cheng, Man Luo, Jianwei Wu, Guo Guo

**Affiliations:** 1 Department of Clinical laboratory, The First People’s Hospital of Guiyang, Guizhou, PR China; 2 School of Basic Medical Sciences, Guizhou Medical University, Guiyang, PR China

**Keywords:** *Musca domestica*, peritrophic matrix protein, RNA interference, intestinal immune, antimicrobial peptide

## Abstract

Peritrophic matrix/membrane (PM) critically prevents the midgut of insects from external invasion by microbes. The proteins in the peritrophic membrane are its major structural components. Additionally, they determine the formation and function of this membrane. However, the role of PM proteins in immune regulation is unclear. Herein, we isolated a novel PM protein (*Md*PM-17) from *Musca domestica* larvae. Further, the function of *Md*PM-17 in regulating host innate immunity was identified. Results showed that the cDNA of *Md*PM-17 full is 635 bp in length. Moreover, it consists of a 477-bp open reading frame encoding 158 amino acid residues. These amino acid residues are composed of two Chitin-binding type-2 domain (ChtBD2) and 19 amino acids as a signal peptide. Moreover, tissue distribution analysis indicates that *Md*PM-17 was enriched expressed in midgut, and moderate levels in the fat body, foregut, and malpighian tubule. Notably, *Md*PM-17 recombinant protein showed high chitin-binding capacity, thus belongs to the Class III PM protein group. *Md*PM-17 protein silencing via RNA interference resulted in the expression of antimicrobial peptide (defensin, cecropins, and diptericin) genes, and this occurred after oral inoculation with exogenous microbes Escherichia coli (Enterobacteriales:Enterobacteriaceae), Staphylococcus aureus (Bacillales:Staphylococcaceae), and Candida albicans (Endomycetales:Saccharomycetaceae)). Therefore, all the antimicrobial peptide (AMP) gene expression levels are high in *Md*PM-17-depleted larvae during microbial infection compared to controls. Consequently, these findings indicate that *Md*PM-17 protein is associated with the antibacterial response from the housefly.

A complex symbiotic relationship exists between an insect and the associated symbiotic/pathogenic microorganisms. This relationship results in a micro-ecological system in the gut of an insect (Hoffmann 2003).The intestinal tract of insects provides a suitable living environment for microorganisms which thereby stimulates the natural immune response by producing antimicrobial peptides (Petridis et al. 2006, Akhtar et al. 2009). Therefore, this inhibits microbial invasion at intestinal epithelium and promotes intestinal homeostasis. In this regard, a better understanding of intestinal innate immunity is essential.

Peritrophic matrix (PM) is a non-cellular structure that surrounds the intestinal epithelium often found in insects (Tellam 1996). It is the first biophysical barrier in the midgut (Lehane 1997, Terra 2001). Additionally, the PM crucially protects the midgut epithelium from mechanical damage through food debris and regulates pathogen infection outcomes (Kuraishi et al. 2011) by preventing microbial invasion. PM is made of proteins, chitin, and other associated components such as proteoglycans. Many PM proteins are highly associated with the chitin through their chitin-binding domains (CBDs) to maintain the stability of PM. Furthermore, they contribute to the penetrability, elasticity, and strength of the PM (Wang and Granados 2001).

PM proteins are classified into four classes based on their tightness level in binding to the PM (Tellam et al. 1999): Class I proteins are removed with physiological buffers and represent loosely associated proteins, likely digestive enzymes and food remnants. Class II proteins are extractible with mild detergents, such as sodium dodecyl sulfate, that disrupt weak ionic interactions, whereas Class III proteins are released only with strong denaturants, such as urea. Class IV proteins cannot be removed by any means and are likely covalently linked to other proteins or chitin. Currently, significant progress has been made in understanding the mechanisms of PM formation and its molecular structure. Additionally, more than 30 PM proteins have been characterized using several insect species including *Trichoplusia ni* (Wang et al. 2004, Guo et al. 2005), *Bombyx mori* (Zhou et al. 2010, Hu et al. 2013), *Plutella xylostella* (Sarauer et al. 2003), *Lucilia cuprina* (Tellam et al. 2003), *Aedes aegypti* (Devenport et al. 2006) among others (Rose et al. 2014, Zhao et al. 2014). A study on a PM protein with CBD structure extracted from a tsetse fly (Hao and Aksoy 2002) reported that it has a role in pathogen growth resistance, therefore suggesting that the PM protein can be a potential congenital immune molecule. Elsewhere, RNA interference (RNAi) was used to silence the expression of two PM proteins in tsetse fly then infected with pathogenic microorganisms (Brian et al. 2014). Results showed that the PM of the flies could regulate the immune response of the intestinal microbes’ ability.

The housefly is a common host for many microorganisms causing bacterial, parasitic, viral, and rickettsia infections (Scott et al. 2014). *Musca domestica* thrives in extreme environments indicating that it has a strong innate immune system. Therefore, it is a commonly used model organism in immunology studies (Tang et al. 2014). In the previous study, we conducted a proteomic analysis targeting PM of housefly larvae (Wang et al. 2016). We further screened one unknown function of PM protein. It was found that *Md*PM-17 protein contains two CBD thus predicting a crucial role in maintaining the stability of PM. Presently, no study has reported characterization or purification of PM proteins from a housefly. In this study, the molecular characteristics of PM proteins were investigated by cloning the *Md*PM-17 gene. Besides, expression analysis of the *Md*PM-17 gene and its recombinant protein was performed. It was thus reported that the functional characterization of *Md*PM-17 proteins suggests an association with immunity from the housefly.

## Materials and Methods

### Insect Rearing and Microbial Strains Used in the Study

The houseflies were bred in a Modern Pathogenic Laboratory, Guizhou Medical University (Guiyang, China). Their larvae were fed with an artificial diet comprising bran and water. They were routinely reared at 26–28°C with 70–80% relative humidity and a photoperiod of 12 h: 12 h (light: dark) for up to the third-instar larval stage (Fu et al. 2009). *Escherichia coli* ATCC 25922 and *Staphylococcus aureus* ATCC 25923 were isolated and orally inoculated to test the innate immune response from the larvae. *Escherichia coli DH5α* was used for regular molecular cloning and BL21/DE3 for protein expression. All bacterial strains were routinely cultured on LB (Luria-Bertani) broth medium. *Candida albicans* ATCC 76615 was cultured in Sabouraud dextrose broth (SDB).

### *Md*PM-17 Gene Expression Analysis Using RT-PCR

Different tissues (fat body, epithelium, foregut, midgut, hindgut, malpighian tubule, and trachea) were dissected from a third-instar stage housefly larvae. Total RNA was extracted from different larval tissues using Trizol reagent (Invitrogen, California, CA). A 2 μg total RNA was synthesized to the cDNA samples through the first-strand cDNA synthesis kit (Takara, Dalian, China). Additionally, the mRNA level of the *Md*PM-17 gene in the third-instar larvae from different tissues was estimated via semi-quantitative RT-PCR. Further, the ribosomal protein S18 (RPS18) was selected as an internal reference, and primers (RPS18F and RPS18R) were used. For the *Md*PM-17 gene, primers (RT-*Md*PM-17F and RT-*Md*PM-17R) were used. The details of primers are displayed in Table 1, which were synthesized and purified by Tianyi-Huiyuan Biotech Co., Ltd. (Beijing,China) at HPLC purification grade.

**Table 1.** Primers used in the experiment

Primers	Sequence (5′–3′)
RT-PCR analysis	
RPS18F	AAGGGTGTGGGTCGCCGTT
RPS18R	GCAATGGGTTGGAGATGAT
RT-*Md*PM-17F	GCAGTTGTCAGACTTAACGAA
RT-*Md*PM-17R	TCACGCATCATCAACACAACCAC-3
cDNA clone	
re-*Md*PM-17F	CGCCATATGGCAGTTGTCAGACTTAACGAAC
re-*Md*PM-17R	AAACTCGAGTCACGCATCATCAACACAACCAC
qRT-PCR analysis	
*Md*PM-17-rt F	CAGCTGCAGCAGTTGTCAGACT
*Md*PM-17-rt R	CCATATTGTTGCCGAGACAGAA
GAPDH-rt F	CAGGAGGCATTGCTGATGAT
GAPDH-rt R	GAAGGCTGGGGCTCATTT
Cecropins-rt F	GGACAAAGTGAAGCTGGATGGTT
Cecropins-rt R	GCTGGGCCACACCAATAGTTTGA
Defensin-rt F	AAATTTCGTCCATGGAGCTGACGC
Defensin-rt R	ACCGCTCAACAAATCGCAAGTAG
Diptericin-rt F	AGTGCAACATTTGTGGTTGCCGAC
Diptericin-rt R	GCCATAACCTGCTGTGGCATCA
RNA interference analysis	
*Md*PM-17-T7 F	TAATACGACTCACTATAGGGGCCACCGTCTCTGGATGTGTT
*Md*PM-17-T7 R	TAATACGACTCACTATAGGGTCACGCATCATCAACACAACCAC
GFP F	ATGGTGAGCAAGGGCGAG
GFP R	TTACTTGTACAGCTCGTCCATGC
GFP-T7 F	TAATACGACTCACTATAGGGATGGTGAGCAAGGGCGAG
GFP-T7 R	TAATACGACTCACTATAGGGTTACTTGTACAGCTCGTCCATGC

### *Md*PM-17 Gene Sequence Analysis and cDNA Cloning

The entire *Md*PM-17 coding region was amplified by PCR using the specific primers re-*Md*PM-17F and re-*Md*PM-17R containing *Nde I* and *Xho I* restriction sites, respectively. The PCR conditions were at 94°C for 3 min, then 35 cycles of 94°C for 30 s, 55°C for 40 s, and 72°C for 4 min with a final extension at 72°C for 10 min. Afterward, the PCR products were inserted into a pET-32a vector and confirmed by DNA sequencing. Sequence features of the *Md*PM-17 gene were analyzed by NCBI blast and ExPASy. The structural domains of the *Md*PM-17 gene were predicted using the Simple Modular Architecture Research Tool (SMART, version 7.0).

### Recombinant *Md*PM-17 Gene Expression and Chitin-Binding Assay

The recombinant plasmid (pET-32a*-Md*PM-17) was transformed into the Transetta strain (DE3). Isopropyl-β-d-thiogalactoside (IPTG) was used to induce protein expression. The *Md*PM-17 recombinant protein was purified by Ni^2+^ affinity chromatography. The chitin-binding capacity of *Md*PM-17 was detected using the chitin-binding assay method (Wang et al. 2004). At first, regenerated chitin for the chitin-binding assay was prepared from chitosan (Sigma, St. Louis, MO). Thereafter, 20 ml of 10% acetic acid were added slowly into one gram chitosan at room temperature and kept overnight. On the next day, 45 ml of methanol were added into the mixture and filtered. The filtrate was stirred on a magnetic stirrer with 1.5 ml acetic anhydride added. Consequently, the mixture gelled and was cut into small pieces. The finely dispersed chitin was washed with water, dried at 80°C, and diluted to 15 mg/ml.

The mixture contained 1 ml *Md*PM-17 protein (100 μg/ml) and 40 mg regenerated chitin at 4°C for 18 h (1 mM ethylenediaminetetraacetic acid [EDTA] and 1 mM phenylmethyl sulfonyl fluoride). The regenerated chitin bound with *Md*PM-17 were washed four times with phosphate-buffered saline (PBS) to remove non-chitin-binding proteins followed by centrifugation. The sediment was incubated with PBS, 0.1M Na_2_CO_3_, 0.5M NaCl, 6 M urea, and 2% SDS, respectively. After 1-h incubation, the supernatants were collected by centrifugations and analyzed by western blot (the pET-32a protein was taken as the negative control).

### Western Blot

Protein samples were resolved by sodium dodecyl sulphate-polyacrylamide gel electrophoresis (SDS–PAGE), and transferred to a PVDF membrane. Membranes were probed using purchased mouse monoclonal anti-His-tag antibody (1:1,000; Roche, USA). Detection of the protein was carried out using horseradish peroxidase conjugated to goat anti-rabbit or goat antimouse secondary antibody (1:5,000; Promega) with the DAB Horseradish Peroxidase Color Development Kit (Beyotime, China).

### RNAi Induced Through Microinjection of dsRNA

The 400 bp for the *Md*PM-17 gene and 705 bp for green fluorescent protein (GFP) genes were amplified using primers containing T7 RNA polymerase promoter by PCR (Table 1). The PCR products served as the template. Further, the dsRNA was synthesized using the MEGAscript RNAi Kit (Life Technologies, Carlsbad, CA). The synthesized dsRNA was then quantified and examined by agarose gel electrophoresis to confirm the expected product size.

Housefly larvae were divided into three groups and injected with *Md*PM-17 dsRNA (*Md*PM-17 RNAi), GFP dsRNA (GFP RNAi), and normal saline (NS), respectively. A total of 2 μg dsRNA were injected per individual larvae. Collection of dsRNA-injected housefly larvae started a day after microinjection and continued for 3 consecutive days (day 1 to day 3 post-injection). Each day, two live ds*Md*PM-17-treated larvae, two live dsGFP-treated larvae, and two live NS-treated larvae were collected for RNA isolation.

Quantitative real-time PCR (qPCR) was used to detect the efficiency of the RNAi. The experiments were repeated three times with *Md*PM-17-rt F/R and GAPDH-rt F/R used as primers. A 20 μl mixture volume (0.8 μl forward primer, 1 μl cDNA template [20 ng/μl], 0.8 μl reverse primer, 10.4μl of SYBR Select Master Mix [Vazyme Biotech, China], and 7 μl of nuclease-free water) was used. The PCR was performed using ABI 7500 Real-Time PCR System at 95°C for 3 min, 40 cycles of 95°C for 10 s, 60°C for 30 s and 72°C for 1 min, and final step at 72°C for 10 min. The relative quantities of *Md*PM-17 transcripts were assessed using the 2^−ΔΔCt^ method.

### Microbial Infection After Silencing of *Md*PM-17 Gene

Bacteria (*E. coli*, *S. aureus*, and *C. albicans*) were orally inoculated to *Md*PM-17-depleted housefly larvae after 24 h of RNAi. A total of 100 larvae were used for each group and were carried out in triplicate. After 6 h, a total of three live larvae were collected for RNA isolation. AMP gene (cecropin, defensin, and diptericin) expression was detected using qPCR.

### Statistical Analysis

The experimental data were analyzed using Student’s *t*-test: *P < 0.05; **P < 0.01; ***P < 0.001, and the values were represented as the mean ± SD. Statistical tests were performed using the Log Rank test within Prism software.

## Results

### Cloning and Characterization of *Md*PM-17 cDNA

**Fig. 1.**
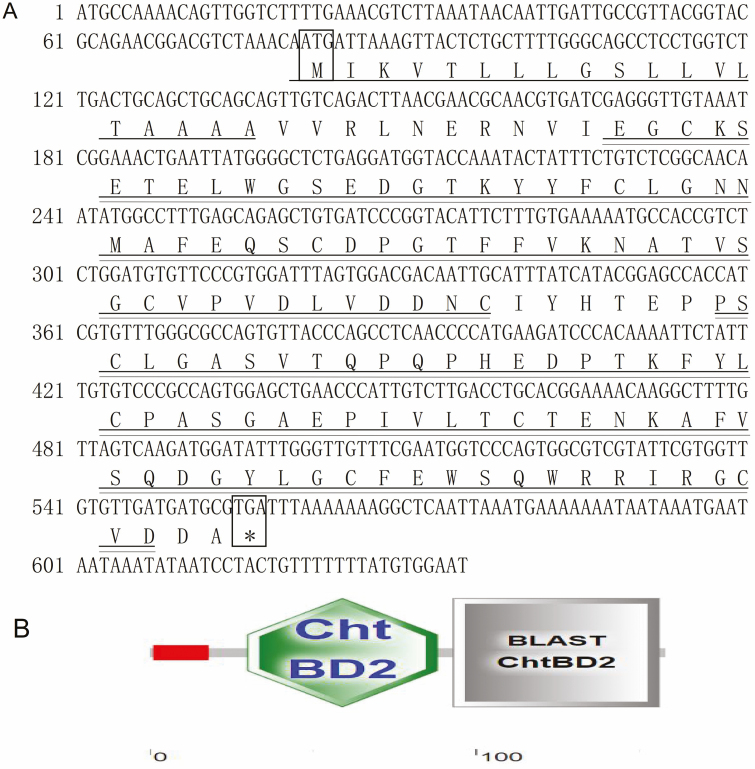
(A) Nucleotide and deduced amino acid sequences of PM protein cDNA *Md*PM-17. The translation initiation codon ATG and stop codon TGA are marked in a box and the predicted signal peptide sequence is underlined. The double underline represents the chitin type II domain. (B) Domains within *Musca domestica* protein of *Md*PM-17.

A full-length cDNA clone was identified and designated as *Md*PM-17. The cDNA is 635 bp in length, containing an ORF of 477 bp, including a 5′untranslated region of 80 bp and a 3′untranslated region of 78 bp (Fig. 1A). The deduced protein sequence showed that *Md*PM-17 was synthesized as a preprotein of 158 amino acid residues with the predicted molecular weight of 17.2976 kDa, pI of 4.55. Results from the SMART database search revealed that the *Md*PM-17 gene carries two chitin-binding type-2 domain (ChtBD2) and putative signal peptide of 19 amino acids (Fig. 1B).

### *Md*PM-17 Gene Expression in Various Tissues

**Fig. 2.**
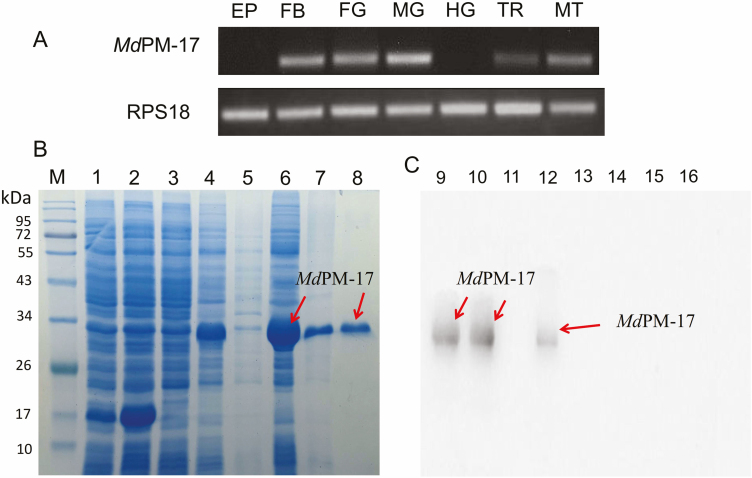
(A) RT-PCR analysis of *Md*PM-17 at different tissues. EP = epidermis; FB = fat body; FG = foregut; MG = midgut; HG = hindgut; TR = trachea; MT = malpighian tubule. (B) SDS–PAGE results of recombinant *Md*PM-17 expression. Lane M: protein marker; Lane 1: the empty vector pET-32a; Lane 2: pET-32a + IPTG; Lane 3: the expression plasmid pET-32a-*Md*PM-17 without IPTG; Lane 4: pET-32a-*Md*PM-17 induced with IPTG; Lane 5: supernatants after disruption of Transetta (DE3) cell from lane 4; Lane 6: sediments of lysate of cell containing pET-32a-*Md*PM-17 induced with IPTG; Lane 7: recombinant protein after renaturation; Lane 8: purified recombinant proteins. (C) Western blot analysis of the chitin-binding activity of recombinant *Md*PM-17 protein with an anti-His antibody; Lane 9: recombinant *Md*PM-17 protein; lane 10: chitin-bound protein MdPM-17; Lane 11: chitin-bound protein pET-32a; Lane 12: 6M Urea; Lane 13: 2% SDS; Lane 14: 0.1 M Na_2_CO_3_; Lane 15: 0.5 M NaCl; Lane 16: PBS.

The expression levels of *the Md*PM-17 gene transcript were studied using RT-PCR. The epidermis, fat body, foregut, midgut, hindgut, malpighian tubule, and trachea were dissected from the third-instar larval stage (Fig. 2A). Results showed that *Md*PM-17 gene had high expression levels in the midgut, and moderate levels in the fat body, foregut, and malpighian tubule. However, there were low expression levels in the trachea.

### Recombinant *Md*PM-17 Gene Expression

The recombinant plasmid (pET-32a-*Md*PM-17) was effectively constructed and transformed into *E. coli.* This was performed with Transetta (DE3) competent cells particularly for expression and protein purification. SDS–PAGE gel (Fig. 2B) separated and purified the recombinant *Md*PM-17 protein successfully.

### Chitin-Binding Capacity by Recombinant *Md*PM-17 Protein

Most PM proteins contain one or more peritrophic domains indicating that they can bind to chitin. In this study, the chitin-binding assay demonstrated that recombinant *Md*PM-17 protein strongly binds to chitin. They can be separated only by 6M urea. Also, the assay indicated that *Md*PM-17 belongs to the class 3 PM protein group (Fig. 2C).

### Regulation of AMP Gene Expression by *Md*PM-17

**Fig. 3.**
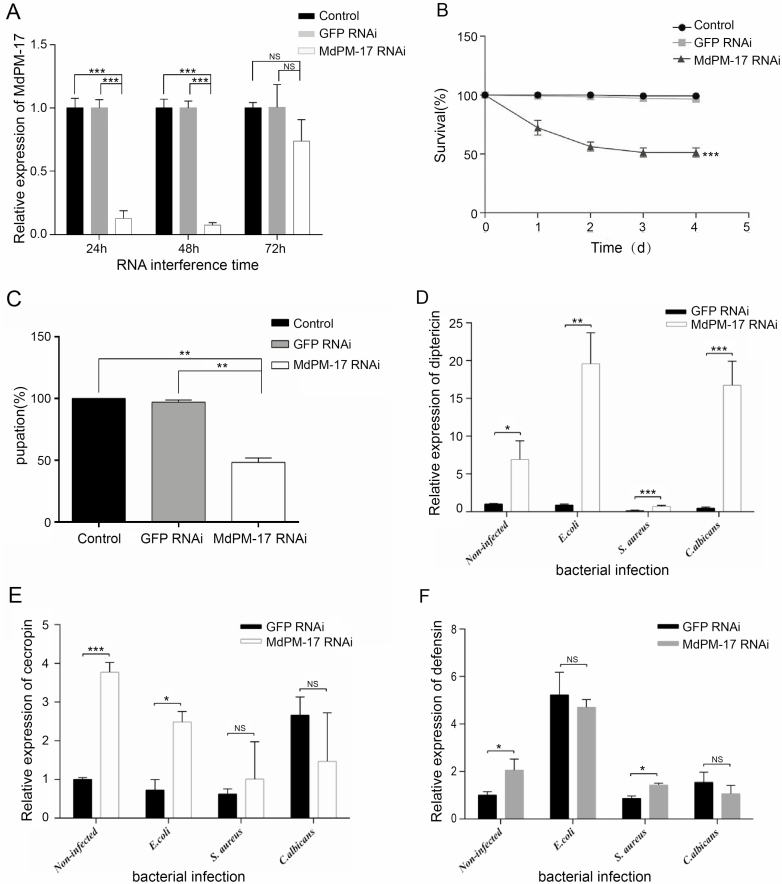
Housefly AMPs expression after RNAi knockdown of *Md*PM-17. (A) Relative gene expression of *Md*PM-17 over time by dsRNA treatment. (B) The survival rate of housefly larvae after RNAi. (C) The pupation rate of housefly larvae after RNAi. (D) Housefly larval diptericin expression after *Md*PM-17 RNAi. (E) Housefly larval cecropins expression after *Md*PM-17 RNAi. (F) Housefly larval defensin expression after *Md*PM-17 RNAi.

The housefly larvae were injected with *Md*PM-17 or GFP dsRNA. The corresponding levels of mRNA transcript in *Md*PM-17 were detected by qPCR. The results showed that *Md*PM-17 was effectively silenced for 24 h in contrast to controls (Fig. 3A). Therefore, *Md*PM-17 transcript levels showed no significant differences between normal and dsGFP treatments. The expression of defensin, cecropins, and diptericin after oral inoculation with exogenous microbes were detected. Results showed that the survival and pupation rate of the *Md*PM-17-depleted larvae was lower than controls (Fig. 3BC).

When housefly larvae are stimulated with *E. coli*, the expression levels of AMP genes (diptericin and cecropins) were high in *Md*PM-17-depleted larvae compared to dsGFP group (Fig. 3D–F). Additionally, there was a significant increase in AMP genes (diptericin and defensin) expression after inoculation with *S. aureus* compared with the dsGFP group (*P* < 0.05). However, inoculation with *C. albicans* showed a significant increase in diptericin expression compared with the dsGFP group (*P* < 0.05).

## Discussion

Intestinal immunity is an important defense system for insects. PM is the first line of defense against intestinal pathogen invasion. Therefore, sufficient information on how precisely PM is associated with the immune response is essential (Kuraishi et al. 2011). Studies in insects have suggested that the PM plays a role in the defense against ingested pathogens. However, most of these studies are based on indirect evidence, such as insect survival analysis after ingestion of corrosive agents that disrupt the PM (Wang and Granados 2000). To date, none of these genes have been studied in the context of the PM, and little is known about the role of mucus and the PM in *M. domestica* larvae gut homeostasis and immunity.

In this study, a cDNA sequence encoding *Md*PM-17 was cloned from *M. domestica.* Thereafter, the protein domain prediction analysis conducted in *Md*PM-17 indicated the presence of two chitin-binding type-2 domains. Consequently, this ChtBD2 domain strongly suggests that *Md*PM-17 protein has a chitin-binding capacity. Moreover, the ChtBD2 structure domain is connected with chitin fiber of PM by a disulphide bond, therefore, contributes to proper structural characteristics, keeps the proteins undamaged by digestive enzymes, and maintains intestinal homeostasis (Felton and Summers 1995).

Results presented in this study indicate that the survival and pupation rate of *Md*PM-17-depleted housefly larvae were severely suppressed compared to the GFP control group. A study by Agrawal silenced a *Tribolium castaneum* PM protein using RNAi, and this led to larval weight loss and increased mortality (Agrawal et al. 2014). Therefore, this suggests that the silencing of the *Md*PM-17 gene might affect the stability of PM and structure of *M. domestica* larvae. It may further interfere with intestinal digestion and absorption hence general larvae growth and development.

Besides, the RNAi experiment indicated that the expression levels of all the three AMPs genes (defensin, diptericin, and cecropins) were high in *Md*PM-17-depleted housefly larvae compared to controls. The increased AMPs gene expression was due to low *Md*PM-17 protein levels in larval PM initiating an immune response from the intestinal epithelium. Therefore, these results show that *Md*PM-17 proteins protect the larvae from intestinal infections by stabilizing the PM. The *Md*PM-17-deficient membrane increases the activity of related antimicrobial peptides against intestinal infection in the larvae. However, different studies indicate that PM promotes infection outcomes by regulating antimicrobial immune mechanisms in *Drosophila* (Wang et al. 2013).

Elsewhere, a study by Kuraishi silenced a *Drosophila* PM protein via gene knockout, and this reduced the thickness of PM making *Drosophila* susceptible to infection. Moreover, high expression levels of antimicrobial peptide were induced suggesting that PM has an effect in limiting intestinal activation and functioning (Kuraishi et al. 2011). The same phenomenon has been reported in the present study. A loss-of-function mutation in the *Md*PM-17 gene results in a reduction of PM width and an increase of its permeability. Upon bacterial ingestion, a higher level of expression of antibacterial peptides was observed in *Md*PM-17 mutants, pointing to an influence of this matrix on bacteria sensing by the intestinal immune pathway.

## Conclusion

This study demonstrated the immunological roles of novel PM protein (*Md*PM-17) in housefly larvae for the first time. Results showed that *Md*PM-17 proteins are associated with larval intestinal immune regulation. Therefore, the novel PM protein (*Md*PM-17) extracted from a housefly provides a foundation for an in-depth understanding of PM biochemical functions and its effects in gut immune responses.
